# The Impact of Chronic Wound Exudate on the Patient, Clinician and Payer: Addressing the Challenges With Foam Dressings

**DOI:** 10.1111/iwj.70369

**Published:** 2025-05-13

**Authors:** Joshua S. Mervis

**Affiliations:** ^1^ Department of Dermatology Tufts Medical Center Boston Massachusetts USA

**Keywords:** chronic wound, foam dressing, quality of life, socioeconomic burden, wound exudate

## Abstract

Chronic wounds are often highly exudative. If not managed properly, chronic wound exudate will result in complications that delay healing and have highly detrimental effects on patients and their carers, as well as add substantial costs to the healthcare system as a whole. This short review article focuses on the role that foam dressings play in achieving moisture balance in the wound while protecting surrounding skin, with emphasis on how their structure and composition contribute to their ability to accomplish these goals. Foam dressing properties can create a conducive environment for wound healing, which ultimately has positive health and economic benefits for patients and society at large. It is hoped that, by reading this article, healthcare professionals will have a better understanding of the role of foam dressings in managing exudate and be better equipped to critique laboratory and clinical studies that have been undertaken to evaluate their performance.

1

**Summary**

- If not managed properly, chronic wound exudate will result in complications that delay healing and increase cost of care.
- Foam dressings play a critical role in achieving moisture balance in the wound while protecting surrounding skin.
- Foam dressing properties can create a conducive environment for wound healing, which ultimately has positive health and economic benefits for patients and society.

## 2 Introduction

Physiologic wound healing consists of four stages (haemostasis, inflammation, proliferation and remodelling) that occur via highly coordinated and complex signalling pathways. Wounds are often classified as either “acute” or “chronic” depending on how efficiently they progress through these stages, though widely adopted formal definitions are lacking. In general, acute wounds progress through the stages in an orderly and timely manner, typically demonstrating greater than 50% reduction in size within 4 weeks [1] By contrast, chronic wounds fail to progress through the normal stages of wound healing and do not heal in a timely fashion, often stalling in the inflammatory phase, taking months or even years to heal. While any type of wound can become chronic, common causes include vascular pathology (i.e., venous and/or arterial) and neurosensory deficits (e.g., diabetic foot ulcers, pressure injury) [2].

## 3 Wound Exudate: Friend or Foe?

The initial inflammatory response to tissue injury and the host response to bacterial load in the wound bed both lead to increased capillary permeability [3, 4]. As the single‐cell capillary walls become stretched, transient gaps are formed between adjacent endothelial cell borders through which fluid leaks into the surrounding tissue and into the wound bed [5].

**FIGURE 1:**
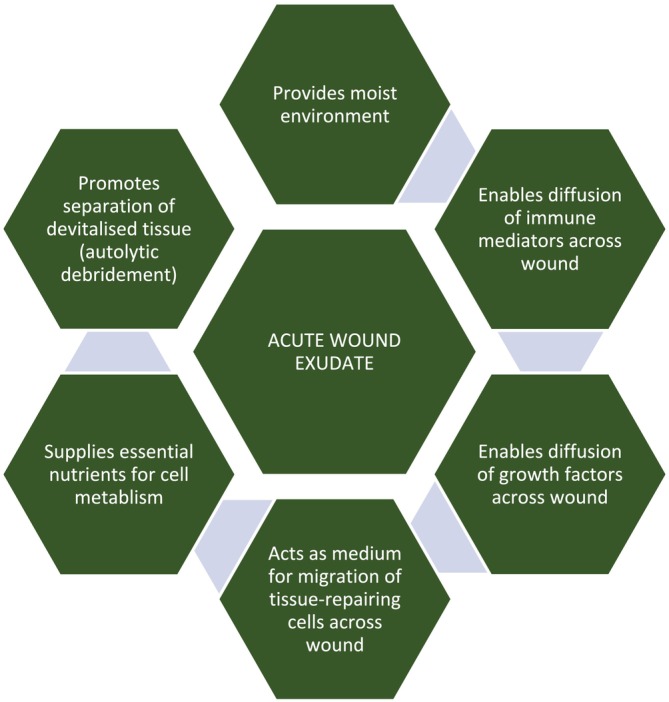
Role of exudate in the healing of acute wounds, as highlighted in a World Union of Wound Healing Societies' consensus document [6].

Wound exudate contains several components including water, glucose, fibrin/fibrinogen, neutrophils, macrophages, platelets, fibroblasts, cytokines, growth factors, matrix metalloproteinases (MMPs), metabolic waste products, microorganisms, and wound debris [3]. In acute wounds, the production of exudate supports the healing process. As detailed in a consensus document published by the World Union of Wound Healing Societies, exudate helps to keep wounds moist, facilitates the removal of devitalised tissue, supplies nutrients for cell metabolism, and provides a medium through which cells, mediators, and growth factors involved in the healing process can diffuse across wounds (Figure 1) [6].

Chronic wounds are often associated with relatively high levels of exudate. In a chronic wound, exudate production may continue to remain high or increase, whereas it generally reduces over time in an acute wound [6]. The heightened and prolonged inflammation associated with chronic wounds is considered a contributor to exudate production, which may be related to wound bioburden [7]. The composition of exudate also differs between acute and chronic wounds, the latter typically containing excessive levels of pro‐inflammatory mediators and activated MMPs that break down the extracellular matrix (Table 1) [2, 6].

**TABLE 1:** Differences in exudate composition between acute and chronic wounds.

Component	Level in chronic wound^a^	Consequence
Inflammatory cytokines	Increased	Heightened matrix metalloprotease activity [5]
Matrix metalloproteases (MMP‐2/MMP‐9)	Increased	Degradation of growth factors and extracellular matrix (ECM); delayed healing; tissue damage (wound/peri‐wound) [5]
Growth factors	Decreased	Reduced proliferation and migration of cells involved in wound healing process [4]
Fibroblasts	Decreased	Reduced ECM production; reduced fibrin breakdown; reduced production of myofibroblasts (which generate contractile forces to heal wounds); reduced angiogenesis [6]

^a^ Relative to acute wound.

## 4 Suboptimal Exudate Management: Clinical, Economic and Quality of Life Implications

To quote a World Union of Wound Healing Societies (WUWHS) consensus document, “Exudate is a normal part of healing; however it can cause problems in the wrong amount, in the wrong place or when of the wrong composition” [6].

**FIGURE 2:**
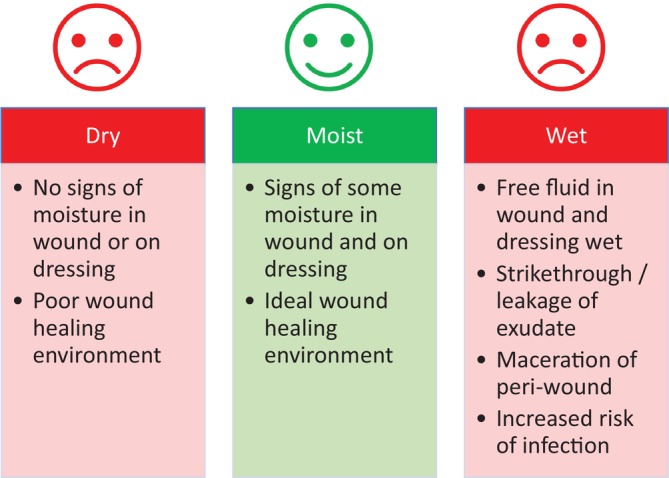
Moisture—getting the balance right for chronic wound management [10, 11].

Current understanding of the ideal wound‐healing environment comes from pivotal research undertaken in the 1960s, when GD Winter found that wounds with a moist surface healed more quickly than those that were scab‐covered and dry [8, 9]. Alternatively, a “wet” wound environment with too much exudate will cause maceration and the breakdown of healthy peri‐wound skin and increase the risk of infection. Thus, the paradigm of moist wound healing was developed and remains key to successful clinical outcomes (Figure 2) [10, 11]. Many chronic wounds get ‘stuck’ in the inflammatory phase of the healing process, with excess exudation leading to delayed or stalled healing, which, in turn, leads to increased demand on clinical resources and financial burden on the patient and society [12].

The leakage of chronic wound exudate onto the peri‐wound region can lead to moisture‐related damage, such as maceration, which also has economic implications for payers. For example, in a study conducted in the United Kingdom in 2014, it was estimated that a single occurrence of maceration had an expected per‐incident cost of £175 (equivalent to £235 for 2024 based on a reputable inflation calculator [13, 14]). Leakage also represents an increased risk of infection by providing a more accessible route for microorganisms to reach the wound [6].

**FIGURE 3:**
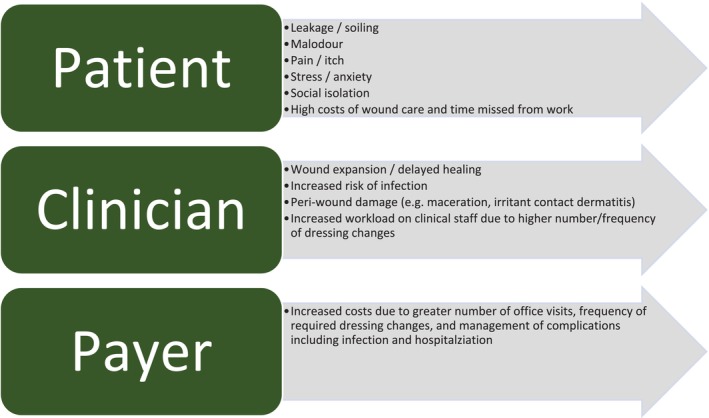
Impact of inadequately managed exuding chronic wounds, a summary of the cited literature [6, 12, 13, 14, 15, 16, 17].

From a patient's and carer's perspective, leakage of exudate and its associated malodour can cause considerable distress. In addition to the burden of increased washing of soiled clothing and bed linen, leakage can adversely affect the psychological wellbeing of patients, leading to feelings of disgust, self‐loathing, and low self‐esteem, as well as withdrawal from activities (work or social) for fear of embarrassment [15, 16]. A high frequency of dressing changes can also be stressful to patients, particularly if dressing changes are time‐consuming or associated with pain and discomfort [17]. The impact of suboptimal exudate management from the perspective of the patient, clinician, and payer, as discussed above, is summarised in Figure 3.

## 5 Dressings to the Rescue!

**FIGURE 4:**
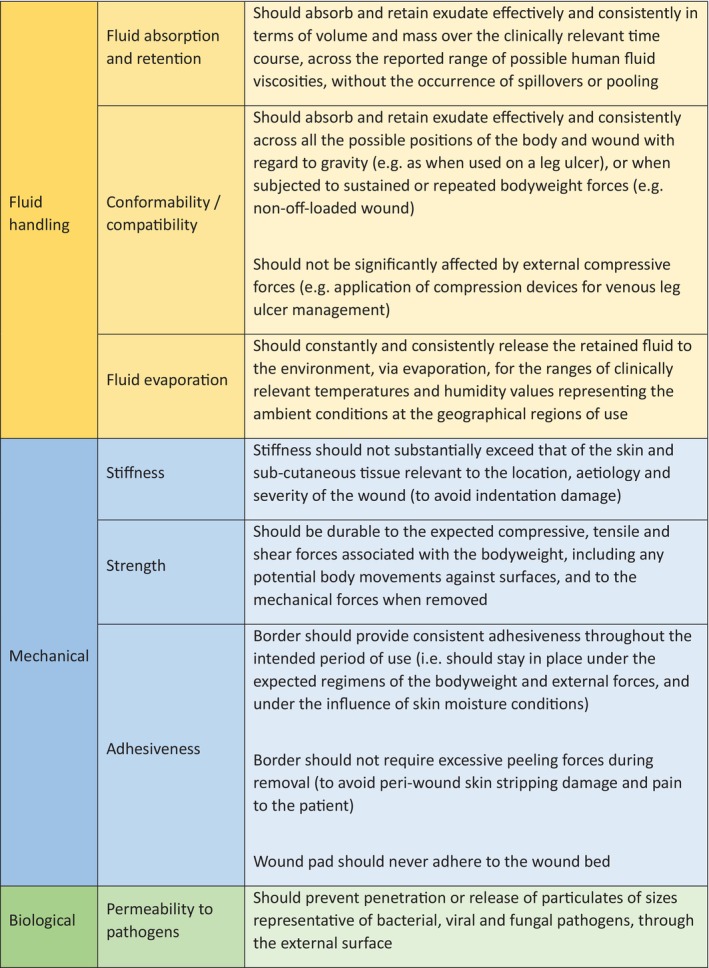
International expert consensus on the basic performance requirements from a foam dressing, [18].

Although negative pressure wound therapy and fluid collection devices are often used to manage highly exudative wounds, absorbent dressings are by far the most common intervention used by clinicians. A variety of different dressing types are used, including those based on foam, gelling fibre, alginate, and superabsorbent polymer technologies (Table 2). Recently, the International Wound Dressing Technology Expert Panel (IWDTEP), a multidisciplinary panel of clinical and scientific experts, convened to discuss and reach consensus on the basic performance requirements that a foam dressing must fulfil in order to most effectively promote wound healing (Figure 4). The panel noted that deviations from these requirements may lead to delays in the healing process and complications that adversely affect the quality of life of patients [18].

**TABLE 2:** Examples of absorbent dressing materials and their roles in exudate management.

Dressing material	Low exudation	Moderate exudation	High exudation
Films (semi‐permeable)	✓		
Hydrogels	✓		
Hydrocolloids	✓	✓	
Foams	✓	✓	✓
Alginates		✓	✓
Fibres		✓	✓
Superabsorbent polymers			✓

*Note:* The properties and intended uses of individual dressings within a dressing material group vary. Other factors, such as wound size, location, and bioburden, will also influence the choice of dressing.

**FIGURE 5:**
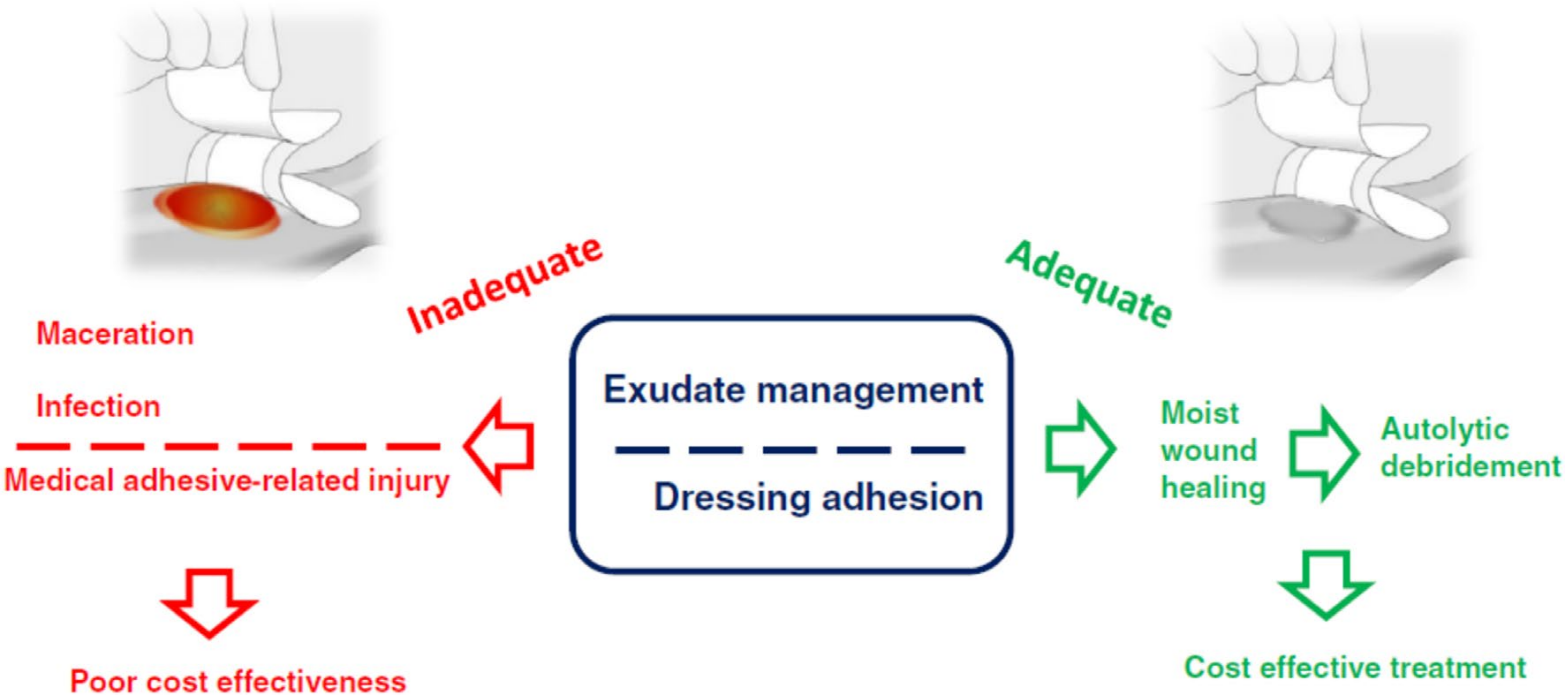
Importance of exudate management and appropriate dressing adhesion to cost‐effective wound care [19].

The aims of exudate management are to optimise wound bed moisture levels, protect the peri‐wound region, manage symptoms, and improve patient quality of life [3]. The fluid handling and adhesive properties of dressings are central to the avoidance of moisture‐related damage to the wound and peri‐wound region, and the maintenance of a moist environment that is conducive to healing (Figure 5) [19].

Foams are a category of absorbent dressing that is designed to manage exudate production and achieve a balance between the extremes of wet and dry; that is, promulgate a moist wound‐healing environment. As is the case for most dressing types, the performance of foam dressings varies significantly because their material, structure, and construction vary across manufacturers [20]. For example, high variabilities within the foam dressing category have been reported in terms of stiffness, strength, conformability, and durability, all of which are crucial for delivering mechanical protection to the wound and peri‐wound region over the intended period of use [21].

Achieving a balance is also important with regard to the adhesive properties of dressings. If dressings incorporate adhesives that are too aggressive, then they are likely to be difficult to remove and subsequently cause trauma to the wound and peri‐wound skin [22, 23]. Furthermore, adhesives are a common cause of both allergic and irritant contact dermatitis, an issue that is often overlooked in wound care but can cause pain, itch, and erosion to the peri‐wound, as well as impair wound healing [24].

As previously mentioned, dressing changes are often stressful for patients, and pain due to the removal of adhesive dressings may be contributory [17]. If, however, dressings utilise adhesive systems that are insufficient, then they are unlikely to stay in place. An unsecured dressing is unlikely to optimally absorb wound exudate and may cause irritation to the underlying tissue from repetitive friction with movement.

The introduction of dressings with soft silicone‐based wound contact surfaces has helped to overcome the problems associated with the aggressive adhesives used in dressings historically. Soft silicone readily adheres to intact dry skin, yet will remain in place on the surface of a moist wound or damaged surrounding skin without adhering to these fragile tissues. Consequently, soft silicone‐coated dressings can be applied and re‐applied without causing damage to the wound or stripping the epidermis in the peri‐wound region [23, 25, 26]. These atraumatic dressings have had a major impact on improving the quality of life of patients by minimising pain and discomfort at dressing changes, while also providing an effective moisture balance [25, 27].

## 6 Concluding Remarks

Foam dressings are well‐established dressings in the armamentarium of interventions used in the management of chronic wounds. Due to their fluid handling and mechanical properties, foam dressings play a key role in maintaining the moist wound‐healing environment. Moreover, foam dressings that incorporate soft silicone‐based wound/skin interfaces can be easily removed without imparting tissue damage to the wound while minimising the risk of peri‐wound irritation and inflammation. Atraumatic removal of such dressings, together with their exudate handling capabilities, gives them the ability to positively impact patients' quality of life not only through advancing wound healing but also by reducing the pain, stress, and stigmatisation of living with a chronic wound.

## Conflicts of Interest

Joshua S. Mervis is a consultant for Mölnlycke Health Care AB and received consultancy fees for work on this article.

## Data Availability

Data sharing not applicable to this article as no datasets were generated or analysed during the current study.
